# Computed tomography staging of colon cancer: improved patient selection for neoadjuvant therapy with combined radiologic tumor and nodal staging

**DOI:** 10.1186/s12885-026-16056-5

**Published:** 2026-04-28

**Authors:** Sonia Lee, Jingjing Yu, Rony Kampalath, Otilio Castillo, Janteshpreet Sandhu, Fatemeh Tajik, Arsha Ostowari, Katharine A. Kirby, Farideh H. Dehkordi-Vakil, Chanon Chantaduly, Peter D. Chang, Valery Vilchez, Richard J. Straker, Matthew D. Whealon, Smitha S. Krishnamurthi, Shishir K. Maithel, Michael J. Stamos, Maheswari Senthil

**Affiliations:** 1 Department of Radiology, University of California, Irvine, Orange, CA 92868 USA; 2 Department of Surgery, University of California, Irvine, 3800 Chapman Ave, Suite 7400, Orange, CA 92868 USA; 3 Center for Statistical Consulting, Department of Statistics, University of California, Irvine, Irvine, CA 92697 USA; 4 Department of Hematology and Oncology, Cleveland Clinic, Cleveland, OH 44195 USA; 5 Department of Surgery, Northwestern University, Chicago, IL 60611 USA

**Keywords:** Colonic neoplasms, Neoadjuvant therapy, Computed tomography, Neoplasm staging, Overstaging rate

## Abstract

**Background:**

Patient selection for neoadjuvant therapy in colon cancer (CC) needs to be improved as utilizing computed tomography (CT) tumor (T) staging alone is associated with overstaging and overtreatment. Therefore, we sought to identify specific nodal imaging features that can be combined with radiologic T staging to improve patient selection.

**Methods:**

Pre-operative CTs of stage I-III CC patients treated with upfront resection (2018–2023) were assessed by an expert abdominal radiologist blinded to the histopathologic staging. Radiologic T and node (N) staging based on five imaging features (single lymph node (LN) > 1 cm, ≥ 3 prominent LNs, irregular borders, heterogenous enhancement, and extramural venous invasion) were compared to the pathologic staging, grouped by proficient or deficient mismatch repair status (pMMR vs. dMMR).

**Results:**

Of the 177 patients, 147 and 30 had pMMR and dMMR CC, respectively. The overstaging rate for pathologic T staging was 6.1% pMMR vs. 6.7% dMMR CC (*p* = 0.91). The overstaging rate for pathologic N disease by any imaging feature was 17.0% pMMR vs. 33.3% dMMR (*p* = 0.04) but improved to 4.8% pMMR vs. 6.7% dMMR (*p* = 0.67) with irregular borders and/or heterogenous enhancement alone. Compared to radiologic T staging only, the addition of N staging (any feature) to T staging resulted in decreased overstaging rates of stage I/low-risk stage II to high-risk stage II/III CC from 25.2% to 15.6% for pMMR CC and 60.0% to 33.3% for dMMR CC. Overstaging rates further decreased to 4.8% for pMMR and 16.7% for dMMR CC with the combination of radiologic T and N staging by irregular borders and/or heterogenous enhancement specifically.

**Conclusion:**

Combining radiologic T and N stage based on any of the five imaging features resulted in lower overstaging rates than radiologic T staging alone, but overstaging rates continued to improve when only irregular borders and heterogenous enhancement were used to assess nodal disease. These results can be used as an initial framework for combining CT T and N staging for CC and improve patient selection for neoadjuvant treatment.

**Supplementary Information:**

The online version contains supplementary material available at 10.1186/s12885-026-16056-5.

## Introduction

Although resectable colon cancer (CC) has been traditionally managed with upfront surgical resection, the concept of neoadjuvant systemic therapy has gained significant attention and momentum due to the recent publications of FOxTROT, NICHE, NeoCol, and OPTICAL trials [1–4]. Selection of patients for participation in neoadjuvant CC trials relies on clinical staging based on radiologic imaging [2, 5].

Computed tomography (CT), magnetic resonance imaging, and positron emission tomography CT scans have been used for CC staging with variable results [6–9]. Since the most common imaging study used for CC staging is a contrast enhanced CT scan, the majority of recent neoadjuvant CC trials used CT imaging for patient selection. In FOxTROT, a multicenter, international, phase III randomized controlled trial, patients with clinical (c) tumor (T)3-T4 resectable CC based on contrast enhanced CT scans were randomized to receive three cycles of neoadjuvant FOLFOX or proceed with upfront surgical resection [1]. In the surgery only arm, 24% of the patients had either stage I or low-risk stage II disease, raising concerns for overtreatment in nearly one-fourth of the enrolled patients [1]. Similarly, the OPTICAL trial enrolled patients with cT3 with ≥ 5 mm extension into adjacent mesenteric fat or cT4 CC and randomized them to receive three months of neoadjuvant FOLFOX or CAPOX chemotherapy vs. upfront surgery. In the upfront surgery arm, 28% of patients either had stage I or stage IIA cancer without any high-risk features [3]. In both these trials, radiologic nodal staging was not used for patient selection. These observations confirm that selection of patients for neoadjuvant systemic therapy based on radiologic T stage alone is associated with a high rate of overstaging, leading to overtreatment of early-stage CC patients who may not require any systemic treatment with upfront surgical resection. Additionally, overstaging has the potential to attenuate treatment effects in a clinical trial due to enrollment of early-stage patients. Therefore, there is a critical need to further refine preoperative radiologic staging with the addition of nodal staging to reduce the risk of overtreatment in neoadjuvant trials targeting high-risk CC (T3N1 or T4) and help avoid toxicity without patient benefit.

However, nodal staging of CC based on CT imaging has been challenging due to low accuracy and inter-reader variability [7, 10–12]. Lymph node (LN) features including size > 1 cm, presence of *≥* 3 prominent LNs, irregular borders, and heterogenous enhancement, as well as poor prognostic features associated with LN disease such as extramural venous invasion (EMVI) [13], have been used to detect node (N) positive disease with varying results [14–16]. In the study conducted to validate the CT methodology for FOxTROT, the specificity of CT to identify malignant LNs based on LN size > 1 cm, presence of *≥* 3 prominent LNs or heterogenous enhancing features was only 42% [17]. In the PRODIGE 22 phase II trial, the overstaging rate of CT to identify N2 disease using > 3 clustered LNs or LNs > 1 cm in shortest diameter was 33% [18]. Other studies, though, report significant associations between the number and location of LNs to malignant nodal status [19]. Furthermore, Rollven et al. found irregular borders and/or heterogenous enhancement to be better at predicting stage III CC compared to criteria based on size and cluster of *≥* 3 LNs [20]. The predictive value of this combination was confirmed in a subsequent study by Rollven et al., with reported sensitivity and specificity of 69% and 100%, respectively [15].

Imaging features associated with the highest accuracy for N positive disease also differ based on proficient vs. deficient mismatch repair (pMMR vs. dMMR) status [21–23]. For example, in a study by Hong et al., internal heterogeneity was a significant independent predictor of LN metastases in pMMR CC while the largest diameter along the short axis of the LNs was significant in dMMR CC [22].

Based on the current evidence, it is abundantly clear that there is lack of consensus about the features or combination of features that have the highest accuracy for detecting nodal disease in CC. Hence, there is a critical need to identify imaging features associated with high specificity for N positive disease in both pMMR and dMMR CC patients. Since the major concern with neoadjuvant treatment in CC is to avoid unnecessary treatment of patients with early-stage disease, it is also essential to identify features associated with the lowest overstaging rates. In this proof-of-concept study, we sought to identify imaging features associated with high specificity for nodal disease based on MMR status and determine if combining radiologic N stage with T stage can reduce the overstaging rate. If combining radiologic T and N staging results in better clinical staging, we aim to create a framework for CC CT staging that incorporates T and N stage, which can then be used to train other radiologists prior to selecting patients for clinical trial participation.

## Methods

### Patient population and data collection

In this retrospective radiology study, we used consecutive sampling to identify adult patients age *≥* 18 years old with stage I-III CC who underwent primary resection at the University of California, Irvine from 2018 to 2023 and had pre-operative intravenous (IV) contrasted CT imaging. Patients with metastatic disease, who received neoadjuvant therapy, had unknown MMR status, or had non-contrasted pre-operative CT imaging were excluded. Patients who did not have an identifiable primary tumor on review of CT imaging by our expert radiologist were also excluded from the analysis. The study was approved by our Institutional Review Board.

Demographic information including age at diagnosis, sex (assigned at birth), and race/ethnicity were collected. Tumor characteristics including pathologic (p)T and pN stage, tumor location, histologic grade, and MMR status were also collected based on pathology reports from the primary resection. Of note, there were 6 patients (3 pMMR and 3 dMMR) with tumor deposits in the pericolic fat confirmed only on pathology review (pN1c disease) who were categorized as pathologic N negative (pN0) disease, as the purpose of the radiologic nodal staging analysis was to test the utility of certain imaging features to detect nodal metastasis specifically. These patients were still categorized as stage III based on American Joint Committee on Cancer (AJCC) staging.

### Imaging analysis

Pre-operative CT abdomen and pelvis scans with IV contrast enhancement, with or without oral contrast, completed either at our institution or from outside imaging centers were included in the analysis. CT studies performed at the study institution were completed on 64–256 slice multidetector CT scanners from GE medical systems (Discovery RX), Phillips (iCT 256, iCT SP), and Siemens (Sensation 64) and reconstructed into slice thickness between 3 and 4 mm. CT studies performed at outside institutions were completed on CT scanners from GE Medical Systems, Phillips, Siemens, Toshiba, and Hitachi, reconstructed into slice thickness between 2 and 5 mm.

**Fig. 1:**
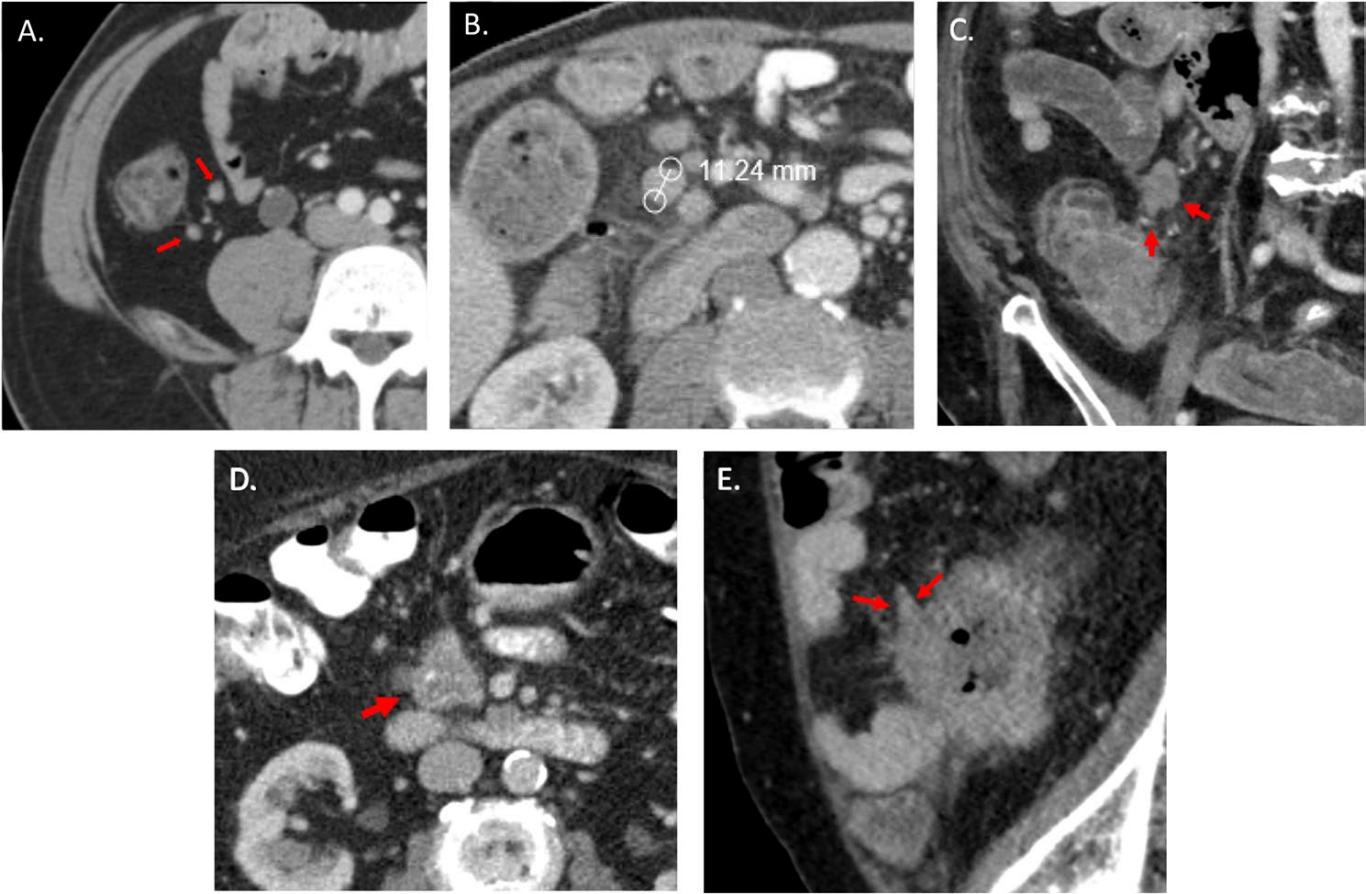
Five imaging features evaluated for nodal disease. **A** Prominent lymph nodes (arrows). **B** Lymph node measuring 1.1 cm (> 1 cm) by the short axis. **C** Lymph node with irregular borders (arrows). **D** Lymph node with heterogenous enhancement (arrow). **E** Primary ascending colon tumor with extramural venous invasion (arrows)

Images were deidentified and reviewed by an expert abdominal radiologist, who was blinded to the histopathologic stage of the patients. Radiologic T and N stage for each patient was recorded. If the tumor invasion depth extended beyond the outer margin of muscularis propria, resulting in focal out-bulging contour beyond the normally smooth and symmetric bowel wall on CT imaging, the tumor was designated as T3. If there was concern for adjacent organ involvement or peritoneal involvement, in addition to tumor extension beyond the serosa, the tumor was categorized as T4. The presence or absence of nodal disease (N0 vs. N1/2) was determined based on the presence or absence of at least one of five imaging features: single LN > 1 cm measured on short axis (S), *≥* 3 prominent LNs (P), irregular LN borders (I), heterogenous enhancement (H), and EMVI (E) (Fig. 1). Prominent LNs were defined as a group of LNs with size > 5 mm by short axis. The short axis was determined as the smallest diameter measured after examining the LN on all planes available (axial, coronal, and sagittal). Data was recorded and managed using REDCap, a secure web-based software platform [24, 25].

### Statistical analysis

Radiologic staging was compared to the histopathologic staging for both T and N disease in pMMR vs. dMMR CC groups. The overstaging rate (false positive (FP)/(true positive (TP)+true negative (TN)+false positive (FP)+false negative (FN)), accuracy ((TP + TN)/(TP + TN+FP + FN)), sensitivity (TP/(TP + FN)), and specificity (TN/(TN + FP)) of each radiologic feature was calculated for pT3/T4 and pN1/N2 disease. Receiver operating characteristic curves were analyzed to yield an area under the curve (AUC).

Inter-reader variability was assessed with CT interpretations by a second abdominal radiologist, read using the same methods described above. Percent agreement and Cohen’s kappa statistic was calculated for T stage, N stage, and overall stage, as well as irregular borders and heterogenous enhancement. Kappa statistic between 0.01 and 0.2 indicates none to slight agreement, 0.21–0.4 indicates fair agreement, 0.41–0.6 indicates moderate agreement, 0.61–0.8 indicates substantial agreement, and 0.81-1.0 indicates almost perfect agreement [26].

Univariable and multivariable logistical regression analyses of the five imaging features to predict pN1/N2 disease were also performed. Based on these results, additional combinations of imaging features to predict positive nodal disease were evaluated. Statistical significance was set as p-value < 0.05. Analyses were performed on StataCorp. 2023. *Stata Statistical Software: Release 18*. College Station, TX: StataCorp.

## Results

### Patient demographics

A total of 177 patients with stage I-III CC treated with primary resection were identified. The majority of patients were male (58.8%) with a median age of 65 years (interquartile range 52–74 years) at diagnosis. The most common race/ethnicity was non-Hispanic white (NHW, 45.2%) (Table 1).

**Table 1:** Demographics and tumor characteristics

	pMMR (*n* = 147)	dMMR (*n* = 30)	*p*-value
Demographic Factors			
Age at diagnosis, median years (IQR)	64 (53–74)	65.5 (51–75)	0.86
Male, n (%)	89 (60.5%)	15 (50.0%)	0.29
Race/Ethnicity, n (%)			0.70
NHW	65 (44.2%)	15 (50.0%)	
Asian	34 (23.1%)	7 (23.3%)	
Hispanic	30 (20.4%)	7 (23.3%)	
Black/AA	2 (1.4%)	0 (0.0%)	
Other/Unknown	16 (10.9%)	1 (3.3%)	
Tumor Characteristics			
Tumor Location, n (%)			0.003
Cecum/Ascending	53 (36.1%)	20 (66.7%)	
Transverse/Hepatic flexure	14 (9.5%)	3 (10.0%)	
Descending/Splenic flexure	14 (9.5%)	4 (13.3%)	
Sigmoid/Rectosigmoid	66 (44.9%)	3 (10.0%)	
Histologic Grade, n (%)			< 0.001
Well/Moderately differentiated	129 (87.8)	18 (60.0%)	
Poorly differentiated	18 (12.2%)	12 (40.0)	
Pathologic Staging, n (%)			
pT3/T4	115 (78.2%)	28 (93.3%)	0.06
pN1/N2*	67 (45.6%)	4 (13.3%)	0.001
AJCC Staging, n (%)			0.002
Stage I	28 (19.0%)	2 (6.7%)	
Low-risk Stage II (T3,N0)	46 (31.3%)	19 (63.3%)	
High-risk Stage II (T4,N0)	3 (2.0%)	2 (6.7%)	
Stage III	70 (47.6%)	7 (23.3%)	
Radiologic Staging, n (%)			
cT3/T4	99 (67.3%)	26 (86.7%)	0.03
cN1/N2	62 (42.2%)	13 (43.3%)	0.91

*pMMR *proficient mismatch repair, *dMMR* deficient mismatch repair, *IQR* Interquartile range, *NHW* Non-Hispanic White, *AA* African American, *p* pathologic, *T* tumor, *N* node, *AJCC* American Joint Committee on Cancer, *c* clinical

*Excludes pN1c patients (pMMR = 3, dMMR = 3) with tumor deposits in pericolic fat only

### Tumor characteristics

Of the 177 patients, 147 patients (83.1%) and 30 patients (16.9%) had pMMR and dMMR CC, respectively. Compared to the pMMR group, the dMMR group had a significantly higher percentage of patients with right-sided tumors (*p* = 0.003) and poorly differentiated cancers (*p* < 0.001). The pMMR group had a lower rate of pT3/T4 disease (78.2% vs. 93.3%, *p* = 0.06) and a significantly higher rate of pN1/N2 disease (45.6% vs. 13.3%, *p* = 0.001) compared to the dMMR group (Table 1).

### Nodal imaging features

The frequency and type of radiologic nodal features detected are shown in Table 2. At least one imaging feature was present in 62/147 (42.2%) pMMR and 13/30 (43.3%) dMMR CC patients (Table 2). For those with pN1/N2 disease specifically, at least one imaging feature was present in 37/67 (55.2%) pMMR CC patients and 3/4 (75.0%) of dMMR CC patients, with EMVI (*n* = 27) and irregular LN borders (*n* = 21) being the most common imaging features (Table 2).

**Table 2:** Frequency and type of nodal imaging features

	Total Cohort		pN1/N2 only	
	pMMR (*n* = 147)	dMMR (*n* = 30)	pMMR (*n* = 67)	dMMR (*n* = 4)
Number of imaging features, *n* (%)				
1	26 (17.7%)	4 (13.3%)	13 (19.4%)	0 (0.0%)
2	25 (17.0%)	4 (13.3%)	15 (22.4%)	1 (25.0%)
3 or more	11 (7.5%)	5 (16.7%)	9 (13.4%)	2 (50.0%)
Type of imaging feature, *n*				
Single LN >1 cm	13	2	9	1
*≥* 3 prominent LNs	28	10	13	1
Irregular LN borders	25	5	18	3
Heterogenous enhancement of LN	8	1	7	1
Extramural venous invasion	39	9	25	2

On univariable logistic regression analysis, single LN > 1 cm (*p* = 0.04), EMVI (*p* = 0.008), irregular LN borders (*p* = 0.001), and heterogenous enhancement (*p* = 0.02) were found to be significant predictors of pathologic nodal disease (Table 3). However, on multivariable logistic regression that included only the imaging features significant on univariable analysis, irregular LN borders alone remained significant (OR 2.86, 95% CI 1.06–7.69, p-value 0.04) (Table 3).

**Table 3:** Logistic regression analysis of five imaging features for predicting pathologic N1/N2 disease

Imaging feature	Univariable			Multivariable*		
	Odds Ratio	95% CI	*p*-value	Odds Ratio	95% CI	*p*-value
Single LN >1 cm (S)	3.31	1.08–10.14	0.04	1.96	0.57–6.81	0.29
*≥* 3 prominent LNs (P)	0.84	0.40–1.76	0.64	---	---	---
Extramural venous invasion (E)	2.48	1.26–4.89	0.008	1.33	0.59–2.99	0.49
Irregular LN borders (I)	4.52	1.93–10.61	0.001	2.86	1.06–7.69	0.04
Heterogenous enhancement of LN (H)	13.33	1.63–109.1	0.02	5.09	0.56–46.5	0.15

*CI * Confidence interval

*Multivariable logistic regression for all imaging features that were significant in univariable logistic regression models

### Nodal imaging feature combinations and overstaging rates

Since irregular borders was the only imaging feature that was statistically significant on multivariable analysis, we performed additional analysis combining irregular borders with the other four imaging features in various combinations, to gain further insights about sensitivity and overstaging rate. The objective of this analysis was to ascertain which combinations provide an optimal balance between sensitivity and overstaging rate. Of the various combinations (Table 4 and Supplemental Table), SPEIH had the highest sensitivity for CT nodal staging in both pMMR (55.2%) and dMMR (75.0%) patients. However, the corresponding overstaging rate for nodal disease was also the highest for both pMMR (17.0%) and dMMR (33.3%) CC. On the other hand, nodal assessment by just irregular borders and/or heterogenous enhancement (IH combination) yielded the lowest sensitivity for pMMR CC (29.9%) but had the lowest overstaging rates for both pMMR (4.8%) and dMMR (6.7%) CC. In the pMMR group, SIH and SI combinations both had overstaging rates of 6.1%. Compared to the IH combination, sensitivities were higher for SIH and SI combinations (37.3% and 35.8%, respectively vs. 29.9%). It is important to note that in the dMMR group, since the number of pathologic LN positive cases was only four and 3/4 cases had irregular borders, the sensitivity remained at 75% with all the combinations. Besides SPEIH, there were no significant differences in overstaging rates between pMMR and dMMR CC for the other feature combinations (Table 4). Additional feature combinations are included in the supplemental table.

**Table 4:** Accuracy and overstaging rates of composite imaging features for radiologic nodal staging

	SPEIH		SIH		SI		IH	
	pMMR (*n* = 62)	dMMR (*n* = 13)	pMMR (*n* = 34)	dMMR (*n* = 6)	pMMR (*n* = 33)	dMMR (*n* = 6)	pMMR (*n* = 27)	dMMR (*n* = 5)
True Positive	37	3	25	3	24	3	20	3
True Negative	55	16	71	23	71	23	73	24
False Positive	25	10	9	3	9	3	7	2
False Negative	30	1	42	1	43	1	47	1
Accuracy	62.6% (54.2%-70.4%)*	63.3% (43.9%-80.1%)	65.3% (57.0%-73.0%)	86.7% (69.3%-96.2%)	64.6% (56.3%-72.3%)	86.7% (69.3%-96.2%)	63.3% (54.9%-71.1%)	90.0% (73.5%-97.9%)
Sensitivity	55.2% (42.6%-67.4%)	75.0% (19.4%-99.4%)	37.3% (25.8%-50.0%)	75.0% (19.4%-99.4%)	35.8% (24.5%-48.5%)	75.0% (19.4%-99.4%)	29.9% (19.3%-42.3%)	75.0% (19.4%-99.4%)
Specificity	68.8% (57.4%-78.7%)	61.5% (40.6%-79.8%)	88.8% (79.7%-94.7%)	88.5% (69.8%-97.6%)	88.8% (79.7%-94.7%)	88.5% (69.8%-97.6%)	91.3% (82.8%-96.4%)	92.3% (74.9%-99.1%)
AUC	0.62 (0.54–0.70)	0.68 (0.42–0.95)	0.63 (0.56–0.70)	0.82 (0.56-1.0)	0.62 (0.56–0.69)	0.82 (0.56-1.0)	0.61 (0.54–0.67)	0.84 (0.59-1.00)
Overstaging rate	17.0% (11.3%-24.1%)	33.3% (17.3%-52.8%)	6.1% (2.8%-11.3%)	10.0% (2.1%-26.5%)	6.1% (2.8%-11.3%)	10.0% (2.1%-26.5%)	4.8% (1.9%-9.6%)	6.7% (0.8%-22.1%)
*p*-value^†^	0.04		0.44		0.44		0.67	

*S* presence of single LN >1 cm, *P* ≥3 prominent LNs, *E * Extravascular venous invasion, *I * Irregular borders, *H * Heterogenous enhancement, *pMMR* proficient mismatch repair, *dMMR* deficient mismatch repair, *AUC* Area under the curve

*95% confidence intervals are provided in parentheses

^†^*p*-value comparing overstaging rate by MMR status

### CT staging accuracy for tumor staging

Next, we analyzed the accuracy of CT staging for T disease as compared to pathologic staging. The accuracy of CT staging to detect pT3/T4 disease was 76.9% for pMMR CC and 80.0% for dMMR CC. The overstaging rate for pathologic T staging was not significantly different between these two groups (6.1% vs. 6.7%, *p* = 0.91) (Table 5).

**Table 5:** Radiologic staging of T3/T4 disease as compared to pathologic T3/T4 disease

	pMMR (*n* = 147)	dMMR (*n* = 30)
True Positive	90	24
True Negative	23	0
False Positive	9	2
False Negative	25	4
Accuracy	76.9% (69.2%-83.4%)*	80.0% (61.4%-92.3%)
Sensitivity	78.3% (69.6%-85.4%)	85.7% (67.3%-96.0%)
Specificity	71.9% (53.3%-86.3%)	0.0%^†^ (0.0-84.2%)
AUC	0.75 (0.66–0.84)	0.43 (0.36–0.50)
Overstaging rate	6.1% (2.8%-11.3%)	6.7% (0.8%-22.1%)
*p*-value^‡^	0.91	

*pMMR * proficient mismatch repair, *dMMR* deficient mismatch repair, *AUC* Area under the curve

*95% confidence intervals are provided in parentheses

^†^Specificity is calculated as true negative/(true negative + false positive). Since the true negative number was 0 in the dMMR group, the specificity was derived as 0

^‡^*p*-value comparing overstaging rate by MMR status

### Impact of adding CT nodal staging to tumor staging on AJCC overstaging rates

We then evaluated CT staging based on the AJCC staging system for CC (Table 6). The risk of overstaging AJCC stage I or low-risk stage II (T3N0) CC to high-risk stage II (T4N0) or stage III CC based on radiologic T3/T4 staging alone was 25.2% in the pMMR group and 60.0% in the dMMR group. The associated sensitivity and specificity were 84.9% and 50% for pMMR CC, and 88.9% and 14.3% for dMMR CC, respectively. When radiologic N stage, as determined by the presence of any of the five imaging features, was combined with radiologic T stage, overstaging rates decreased to 15.6% for pMMR and 33.3% for dMMR CC. However, when the radiologic N stage based on irregular border and heterogenous enhancement was combined with the radiologic T stage, the overstaging rate further decreased to 4.8% for pMMR and 16.7% for dMMR patients. As shown in Table 6, the sensitivity for SPEIH was higher than IH in both cohorts (pMMR 57.5% vs. 34.2%; dMMR 55.6% vs. 44.4%). Compared to dMMR CC, the overstaging rates for pMMR CC were significantly lower for AJCC staging (Table 6).

**Table 6:** Accuracy and overstaging rates for American Joint Committee on Cancer stage I/low-risk II to high-risk stage II/III colon cancer

	Radiologic T stage alone		Radiologic T stage and N stage based on SPEIH		Radiologic T stage and N stage based on IH	
	pMMR (*n* = 147)	dMMR (*n* = 30)	pMMR (*n* = 147)	dMMR (*n* = 30)	pMMR (*n* = 147)	dMMR (*n* = 30)
True Positive	62	8	42	5	25	4
True Negative	37	3	51	11	67	16
False Positive	37	18	23	10	7	5
False Negative	11	1	31	4	48	5
Accuracy	67.3% (59.1%-74.8%)*	36.7% (19.9%-56.1%)	63.3% (54.9%-71.1%)	53.3% (34.3%-71.7%)	62.6% (54.2%-70.4%)	66.7% (47.2%-82.7%)
Sensitivity	84.9% (74.6%-92.2%)	88.9% (51.8%-99.7%)	57.5% (45.4%-69.0%)	55.6% (21.2%-86.3%)	34.2% (23.5%-46.3%)	44.4% (13.7%-78.8%)
Specificity	50.0% (38.1%-61.9%)	14.3% (3.1%-36.3%)	68.9% (57.1%-79.2%)	52.4% (29.8%-74.3%)	90.5% (81.5%-96.1%)	76.2% (52.8%-91.8%)
AUC	0.70 (0.62–0.77)	0.53 (0.34–0.72)	0.63 (0.55–0.71)	0.53 (0.34–0.72)	0.68 (0.60–0.75)	0.60 (0.41–0.77)
Overstaging rate	25.2% (18.4%-33.0%)	60.0% (40.6%-77.3%)	15.6% (10.2%-22.5%)	33.3% (17.3%-52.8%)	4.8% (1.9%-9.6%)	16.7% (5.6%-34.7%)
*p*-value^†^	< 0.001		0.02		0.02	

*S* presence of single LN >1 cm, *P* ≥3 prominent LNs, *E* Extravascular venous invasion, *I* Irregular borders, *H* Heterogenous enhancement, *pMMR* proficient mismatch repair, *dMMR* deficient mismatch repair, *AUC* Area under the curve

^*^95% confidence intervals are provided in parentheses

^†^*p*-value comparing overstaging rate by MMR status

### Inter-reader variability

Finally, inter-reader variability was assessed with a second abdominal radiologist. Percent agreement and Cohen’s kappa was 80.8% and 0.53 for T3/T4 disease, 80.8% and 0.60 for N1/N2 disease and 73.1% and 0.45 for high-risk stage II/III disease. Inter-reader variability was also assessed for irregular borders and heterogenous enhancement, as those two imaging features together produced the lowest overstaging rates. Percent agreement and Cohen’s kappa was 84.6% and 0.42 for irregular borders and 92.3% and 0.47 for heterogenous enhancement.

## Discussion

Here, we present an initial proof-of-concept study of CT CC staging using radiologic T and N staging for both pMMR and dMMR CC. Our study provides several key findings that are extremely relevant in the current landscape of neoadjuvant treatment in CC. First, we show that the overstaging rate of pN1/N2 disease varies based on the type and combination of imaging features, with the combination of irregular borders and heterogenous enhancement producing the lowest overstaging rate for both pMMR and dMMR CC. Second, we show that although the likelihood of radiologic T stage to overstage pT1/T2 tumors to T3/T4 disease is around 6% for both pMMR and dMMR CC, the likelihood of overstaging AJCC stage I/low-risk II to high-risk stage II/III with radiologic T stage alone was as high as 25.2% in pMMR and 60% in dMMR CC. Finally, we showed that when radiologic N stage based on an any imaging feature was combined with radiologic T stage, the AJCC overstaging rate decreased to 15.6% in pMMR CC and 33.3% in dMMR CC. These overstaging rates further improved to 4.8% and 16.7%, respectively, with irregular borders and heterogenous enhancement alone.

This data provides a major framework for radiologic CT staging of CC to decrease overstaging rates. One of the major criticisms of the FOxTROT trial is that nearly 24% of the patients in the neoadjuvant arm may have been overstaged and over treated [1]. Other neoadjuvant treatment trials, namely OPTICAL and PRODIGE 22, have reported even higher overstaging rates of 28% and 33%, respectively [3, 18]. Interestingly, the results of overstaging in our study with radiologic T stage alone were similarly high (25.2%). However, when radiologic T and N stage were combined, the overstaging rate dropped for both pMMR and dMMR groups. This is an important finding and incorporating both radiologic T and N disease for patient selection should be duly considered when designing neoadjuvant treatment trials in CC.

Although irregular borders was the only imaging feature significant on multivariable analysis for predicting pathologic nodal disease, we recognize the possible limitations to staging nodal disease based on only one imaging feature. Hence, we performed a comprehensive analysis of sensitivity, specificity, and overstaging rate for various feature combinations that included irregular borders, providing valuable information that could be used for the selection of imaging features based on the needs of a clinical trial.

In general, there was an inverse relationship between sensitivity and overstaging rates. In the pMMR group, the sensitivity decreased from 55.2% to 29.9% while overstaging improved from 17.0% to 4.8% across the various combinations from SPEIH to IH, respectively. It is important to note that sensitivity improved when combining N stage using IH features with radiologic T stage to detect high-risk stage II/III CC patients while still yielding acceptable overstaging rates. Nevertheless, low sensitivity may not be acceptable for certain clinical trials as it would exclude a majority of patients who may be eligible for a neoadjuvant treatment trial. Hence, evaluating alternate combinations such as SIH and SI that provides a reasonable balance between sensitivity and overstaging rate may be more desirable than the IH combination. Depending on the treatment being studied in a clinical trial and level of tolerance for overstaging compared to understaging, investigators may choose between different combinations of imaging features.

Furthermore, since pMMR and dMMR CC are biologically different, we investigated potential differences in accuracy and overstaging rates based on MMR status [27]. We observed the overstaging rate for T stage was similar for pMMR CC (6.1%) compared to dMMR CC (6.7%). We also observed overstaging rates for N stage were lower for pMMR CC than dMMR CC across different imaging combinations. Overall, our reported overstaging rates for both T and N staging were lower than those reported in a study by Linhares et al. investigating the accuracy of CT staging for pMMR vs. dMMR CC [28].

We acknowledge, however, the small number of dMMR CC patients included in our cohort (16.9%) as a major limitation to our study. Furthermore, only 4 out of 30 (13.3%) dMMR CC patients had pathologic nodal disease. Although our cohort reflects the reported 15% prevalence of dMMR CC and lower LN involvement compared to pMMR CC [29–31], it is important to note the limited statistical power of our results in comparison to pMMR CC and view this analysis as more exploratory than confirmatory. Further validation studies that include larger numbers of dMMR patients should be performed.

Another major limitation is this is a single institution study from an academic center with expert radiologists reading the CT scans. Consequentially, the accuracy of CT staging was high, and the inter-reader variability showed moderate to substantial agreement for T and N staging. However, since experience of radiologist is a significant predictor of CT staging accuracy [32], generalizability of these findings to a wide range of practice settings needs to be further validated. Nevertheless, our study primarily serves as proof-of-concept that highlights specific imaging features associated with nodal positivity and the feasibility of combining radiologic T and N stage for CC staging. The results of this study are intended to serve as a framework to train radiologists and perform further validation studies to the test the reproducibility and generalizability of our results. With the appropriate level of training and experience, CT imaging could serve as a reliable diagnostic tool to accurately identify patients with CC for neoadjuvant clinical trials.

## Conclusion

Overstaging rates based on CT T staging alone are high. However, combining radiologic N staging with T staging is associated with lower overstaging rates in both pMMR and dMMR CC. Collectively, these results demonstrate the feasibility of utilizing CT T and N staging to clinically stage CC with low overstaging rates and better select patients for neoadjuvant therapy.

## Supplementary Information

Supplementary Material 1.

## Data Availability

The datasets used and/or analyzed during the current study are available from the corresponding author on reasonable request.

## Abbreviations

CC: Colon cancer
CT: Computed tomography
T: Tumor
N: Node
LN: Lymph node
c: Clinical
p: Pathologic
pMMR: Proficient mismatch repair
dMMR: Deficient mismatch repair
IV: Intravenous
S: Single lymph node > 1 cm
P: ≥ 3 prominent lymph nodes
I: Irregular lymph node borders
H: Heterogenous enhancement
EMVI: Extramural venous invasion
AJCC: American Joint Committee on Cancer
TP: True positive
TN: True negative
FP: False positive
FN: False negative
AUC: Area under the curve
