# Supplementary material for: Computed tomography staging of colon cancer: improved patient selection for neoadjuvant therapy with combined radiologic tumor and nodal staging
Source: BMC Cancer. 2026 Apr 28;26:744. doi: 10.1186/s12885-026-16056-5 (PMC13255333; doi:10.1186/s12885-026-16056-5)
Supplement: Supplementary file 1 — Supplementary Material 1. [file 12885_2026_16056_MOESM1_ESM.docx]

**Supplemental Table: Accuracy and overstaging rates of additional composite imaging features for radiologic nodal staging**

|  | **PEIH** | | **SEIH** | | **SPEI** | |
| --- | --- | --- | --- | --- | --- | --- |
|  | **pMMR**  **(n=61)** | **dMMR**  **(n=13)** | **pMMR**  **(n=51)** | **dMMR**  **(n=10)** | **pMMR**  **(n=62)** | **dMMR**  **(n=13)** |
| True Positive | 36 | 3 | 32 | 3 | 38 | 3 |
| True Negative | 55 | 16 | 61 | 19 | 53 | 13 |
| False Positive | 25 | 10 | 19 | 7 | 24 | 10 |
| False Negative | 31 | 1 | 35 | 1 | 32 | 4 |
| Accuracy | 61.9%  (53.5%-69.8%) | 63.3%  (43.9%-80.1%) | 63.3%  (54.9%-71.1%) | 73.3%  (54.1%-87.7%) | 61.9%  (53.5%-69.8%) | 53.3%  (34.3%-71.7%) |
| Sensitivity | 53.7%  (41.1%-66.0%) | 75.0%  (19.4%-99.4%) | 47.8%  (35.4%-60.3%) | 75.0%  (19.4%-99.4%) | 54.3%  (41.9%-66.3%) | 42.9%  (9.9%-81.6%) |
| Specificity | 68.8%  (57.4%-78.7%) | 61.5%  (40.6%-79.8%) | 76.3%  (65.4-85.1%) | 73.1%  (52.2%-88.4%) | 68.8%  (57.3%-78.9%) | 56.5%  (34.5%-76.8%) |
| AUC | 0.61  (0.53-0.69) | 0.68  (0.42-0.95) | 0.62  (0.54-0.70) | 0.74  (0.48-1.0) | 0.62  (0.54-0.69) | 0.50  (0.27-0.72) |
| **Overstaging rate** | 17.0%  (11.3%-24.1%) | 33.3%  (17.3%-52.8%) | 12.9%  (8.0%-19.4%) | 23.3%  (9.9%-42.3%) | 16.3%  (10.7%-23.3%) | 33.3%  (17.3%-52.8%) |
| **p-value**^†^ | 0.04 | | 0.14 | | 0.03 | |
|  | **SPIH** | | **EIH** | | **SPI** | |
|  | **pMMR**  **(n=53)** | **dMMR**  **(n=12)** | **pMMR**  **(n=46)** | **dMMR**  **(n=10)** | **pMMR**  **(n=52)** | **dMMR**  **(n=12)** |
| True Positive | 33 | 3 | 29 | 3 | 32 | 3 |
| True Negative | 57 | 14 | 63 | 19 | 57 | 14 |
| False Positive | 20 | 9 | 17 | 7 | 20 | 9 |
| False Negative | 37 | 4 | 38 | 1 | 38 | 4 |
| Accuracy | 61.2%  (52.8%-69.1%) | 56.7%  (37.4%-74.5%) | 62.6%  (54.2%-70.4%) | 73.3%  (54.1%-87.7%) | 60.5%  (52.2%-68.5%) | 56.7%  (37.4%-74.5%) |
| Sensitivity | 47.1%  (35.1%-59.4%) | 42.9%  (9.9%-81.6%) | 43.3%  (31.2%-56.0%) | 75.0%  (19.4-99.4%) | 45.7%  (33.7%-58.1%) | 42.9%  (9.9%-81.6%) |
| Specificity | 74.0%  (62.8%-83.4%) | 60.9%  (38.5%-80.3%) | 78.8%  (68.2%-87.1%) | 73.1%  (52.2%-88.4%) | 74.0%  (62.8%-83.4%) | 60.9%  (38.5%-80.3%) |
| AUC | 0.61  (0.53-0.68) | 0.52  (0.30-0.74) | 0.61  (0.54-0.69) | 0.74  (0.48-1.0) | 0.60  (0.52-0.68) | 0.52  (0.30-0.74) |
| **Overstaging rate** | 13.6%  (8.5%-20.2%) | 30.0%  (14.7%-49.4%) | 11.6%  (6.9%-17.9%) | 23.3%  (9.9%-42.3%) | 13.6%  (8.5%-20.2%) | 30.0%  (14.7%-49.4%) |
| **p-value**^†^ | 0.03 | | 0.09 | | 0.03 | |
|  | **PEI** | | **PIH** | | **SEI** | |
|  | **pMMR**  **(n=61)** | **dMMR**  **(n=13)** | **pMMR**  **(n=51)** | **dMMR**  **(n=12)** | **pMMR**  **(n=51)** | **dMMR**  **(n=10)** |
| True Positive | 37 | 3 | 31 | 3 | 32 | 3 |
| True Negative | 53 | 13 | 57 | 14 | 58 | 16 |
| False Positive | 24 | 10 | 20 | 9 | 19 | 7 |
| False Negative | 33 | 4 | 39 | 4 | 38 | 4 |
| Accuracy | 61.2%  (52.8%-69.1%) | 53.3%  (34.3%-71.7%) | 59.9%  (51.5%-67.9%) | 56.7%  (37.4%-74.5%) | 61.2%  (52.8%-69.1%) | 63.3%  (43.9%-80.1%) |
| Sensitivity | 52.9%  (40.6%-64.9%) | 42.9%  (9.9%-81.6%) | 44.3%  (32.4%-56.7%) | 42.9%  (9.9%-81.6%) | 45.7%  (33.7%-58.1%) | 42.9%  (9.9%-81.6%) |
| Specificity | 68.8%  (57.3%-78.9%) | 56.5%  (34.5%-76.8%) | 74.0%  (62.8%-83.4%) | 60.9%  (38.5%-80.3%) | 75.3%  (64.2%-84.4%) | 69.6%  (47.1%-86.8%) |
| AUC | 0.61  (0.53-0.69) | 0.50  (0.27-0.72) | 0.59  (0.52-0.67) | 0.52  (0.30-0.74) | 0.61  (0.53-0.68) | 0.56  (0.34-0.78) |
| **Overstaging rate** | 16.3%  (10.7%-23.3%) | 33.3%  (17.3%-52.8%) | 13.6%  (8.5%-20.2%) | 30.0%  (14.7%-49.4%) | 12.9%  (8.0%-19.4%) | 23.3%  (9.9%-42.3%) |
| **p-value**^†^ | 0.03 | | 0.03 | | 0.14 | |
|  | **PI** | | **EI** | |  |  |
|  | **pMMR**  **(n=49)** | **dMMR**  **(n=12)** | **pMMR**  **(n=46)** | **dMMR**  **(n=10)** |  |  |
| True Positive | 29 | 3 | 29 | 3 |  |  |
| True Negative | 57 | 14 | 60 | 16 |  |  |
| False Positive | 20 | 9 | 17 | 7 |  |  |
| False Negative | 41 | 4 | 41 | 4 |  |  |
| Accuracy | 58.5%  (50.1%-66.6%) | 56.7%  (37.4%-74.5%) | 60.5%  (52.2%-68.5%) | 63.3%  (43.9%-80.1%) |  |  |
| Sensitivity | 41.4%  (29.8%-53.8%) | 42.9%  (9.9%-81.6%) | 41.4%  (29.8%-53.8%) | 42.9%  (9.9%-81.6%) |  |  |
| Specificity | 74.0%  (62.8%-83.4%) | 60.9%  (38.5%-80.3%) | 77.9%  (67.0%-86.6%) | 69.6%  (47.1%-86.8%) |  |  |
| AUC | 0.58  (0.50-0.65) | 0.52  (0.30-0.74) | 0.60  (0.52-0.67) | 0.56  (0.34-0.78) |  |  |
| **Overstaging rate** | 13.6%  (8.5%-20.2%) | 30.0%  (14.7%-49.4%) | 11.6%  (6.9%-17.9%) | 23.3%  (9.9%-42.3%) |  |  |
| **p-value**^†^ | 0.03 | | 0.09 | |  |  |

*95% confidence intervals are provided in parentheses

^†^p-value comparing overstaging rate by MMR status

^‡^S - presence of single LN >1cm, P - >3 prominent LNs, E - extravascular venous invasion, I - irregular borders, H - heterogenous enhancement, pMMR - proficient mismatch repair, dMMR - deficient mismatch repair, AUC - area under the curve
